# Inhibition of PDE5A1 guanosine cyclic monophosphate (cGMP) hydrolysing activity by sildenafil analogues that inhibit cellular cGMP efflux

**DOI:** 10.1111/jphp.12693

**Published:** 2017-02-17

**Authors:** Anna Subbotina, Aina W. Ravna, Roy A. Lysaa, Ruben Abagyan, Ryszard Bugno, Georg Sager

**Affiliations:** ^1^ Experimental and Clinical PharmacologyDepartment of Medical BiologyFaculty of Health SciencesUniversity of Tromsø – The Arctic University of NorwayTromsøNorway; ^2^ Skaggs School of Pharmacy and Pharmaceutical SciencesUniversity of California – San DiegoLa JollaCAUSA; ^3^ Department of Medicinal ChemistryInstitute of PharmacologyPolish Academy of SciencesKrakówPoland

**Keywords:** guanosine cyclic monophosphate, inhibitors, molecular modelling, PDE5, sildenafil analogues

## Abstract

**Objectives:**

To determine the ability of 11 sildenafil analogues to discriminate between cyclic nucleotide phosphodiesterases (cnPDEs) and to characterise their inhibitory potencies (*K*_i_ values) of PDE5A1‐dependent guanosine cyclic monophosphate (cGMP) hydrolysis.

**Methods:**

Sildenafil analogues were identified by virtual ligand screening (VLS) and screened for their ability to inhibit adenosine cyclic monophosphate (cAMP) hydrolysis by PDE1A1, PDE1B1, PDE2A1, PDE3A, PDE10A1 and PDE10A2, and cGMP hydrolysis by PDE5A, PDE6C, PDE9A2 for a low (1 nm) and high concentration (10 μm). Complete IC_50_ plots for all analogues were performed for PDE5A‐dependent cGMP hydrolysis. Docking studies and scoring were made using the ICM molecular modelling software.

**Key findings:**

The analogues in a low concentration showed no or low inhibition of PDE1A1, PDE1B1, PDE2A1, PDE3A, PDE10A1 and PDE10A2. In contrast, PDE5A and PDE6C were markedly inhibited to a similar extent by the analogues in a low concentration, whereas PDE9A2 was much less inhibited. The analogues showed a relative narrow range of *K*_i_ values for PDE5A inhibition (1.2–14 nm). The sildenafil molecule was docked in the structure of PDE5A1 co‐crystallised with sildenafil. All the analogues had similar binding poses as sildenafil.

**Conclusions:**

Sildenafil analogues that inhibit cellular cGMP efflux are potent inhibitors of PDE5A and PDE6C.

## Introduction

Cyclic nucleotide signalling plays an essential role in normal cell physiology and is impaired in many pathological conditions, such as heart disease, pulmonary hypertension, chronic obstructive pulmonary disease, obesity, diabetes and cancer.^1^ The family of human phosphodiesterases (PDEs) comprises 11 main forms, from which PDEs 4, 7 and 8 are adenosine cyclic monophosphate (cAMP) selective; PDEs 5, 6 and 9 are guanosine cyclic monophosphate (cGMP) selective; and PDEs 1, 2, 3, 10 and 11 hydrolyse both cAMP and cGMP.^2^ However, sildenafil raises cellular cGMP levels by two mechanisms, reduction in cellular efflux by ATP‐binding cassette transporter subfamily C, member 5 (ABCC5), previously termed multidrug resistance‐associated protein 5 (MRP5),^3^ in addition to inhibition of PDE5 activity.^4^

Observations suggest that some binding site resemblance exists between PDE5 and ABCC5. In addition to sildenafil, other compounds with ability to inhibit PDE5 activity also reduce cellular cGMP efflux, such as zaprinast,^3^, ^5^, ^6^ dipyridamole,^5^, ^6^ vardenafil and tadalafil^6^ and trequinsin.^3^ In contrast, non‐selective PDE inhibitors, such as IBMX (3‐isobutyl‐1‐methyl‐xanthine),^6^, ^7^ caffeine and theophylline,^6^ have much lower affinity for the cGMP efflux pump.

The *K*_i_ ratio for sildenafil inhibition of cellular cGMP efflux (ABCC5) and hydrolysis (PDE5) is approximately 1000 : 1. In an attempt to balance the action on ABCC5 and PDE5 (*K*_i_ ratio reduction), we identified a series of 11 high‐affinity cGMP transporter inhibitors by virtual ligand screening (VLS).^8^ Some of them, IN‐01 and IN‐02 with *K*_i_ values of 75 and 65 nm, respectively, were clearly more potent than sildenafil (*K*_i_ of 1200 nm) in their inhibition of cGMP efflux.^8^ The present work characterises their selectivity towards other cnPDEs, their interaction with PDE5A determined both by inhibition of cGMP hydrolysis, and docking studies of the analogues into the enzyme‐binding site. The possibility of creating dual and balanced inhibitors (of both PDE5 and ABCC5) by VLS (virtual ligand screening) represents the novelty of this study.

## Materials and Methods

### Sildenafil analogues

The sildenafil analogues (Table 1) were purchased from Ambinter (Greenpharma SAS, Orleans, France) with exception of 4‐ethoxy‐3‐(1‐methyl‐7‐oxo‐3‐propyl‐4H‐pyrazolo[4, 3‐d]pyrimidine‐5‐yl)‐*N*‐[3‐(1‐methylpyrrolidin‐2‐yl)pyridine‐2‐yl] benzenesulfonamide (I‐03) which was unavailable. Consequently, this compound was synthesised at the Department of Medicinal Chemistry, Institute of Pharmacology, Polish Academy of Sciences, Kraków, Poland. The synthesis was achieved, using commercially available 5‐(2‐ethoxyphenyl)‐1‐methyl‐3‐*n*‐propyl‐1,6‐dihydro‐7H‐pyrazolo[4,3‐d]‐7‐pyrimidinone (Sigma‐Aldrich, Schnelldorf, Germany), in three‐step sequence following procedures reported in the literature.^9^ 2‐Aminonicotine used in the last step was prepared from (−)‐nicotine according to the previously published procedure.^10^

**Table 1:**
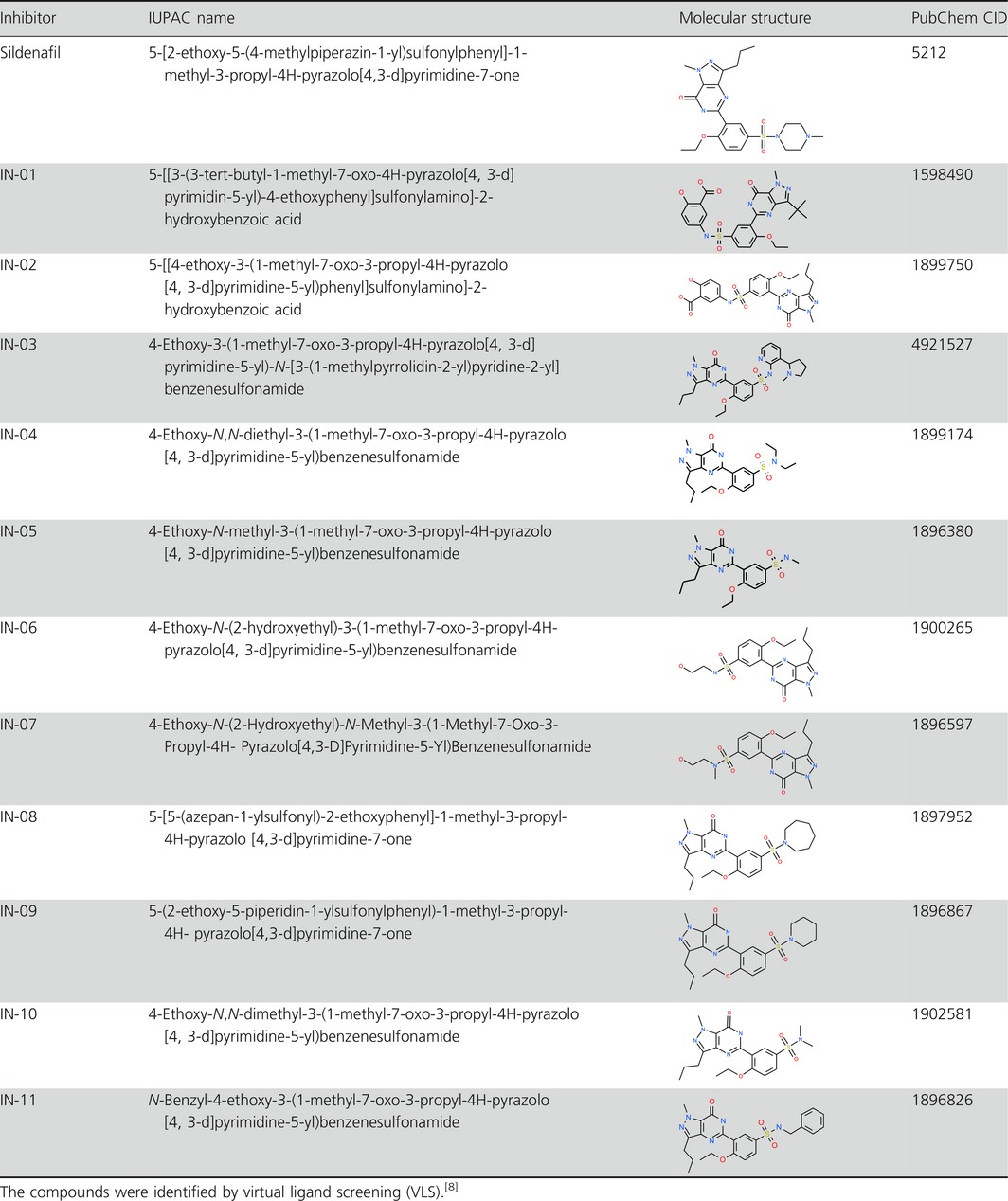
Inhibitors (sildenafil analogues), IUPAC‐names, molecular structure and PubChem CID

### Phosphodiesterase assay for screening of sildenafil analogues

The screening was performed by BPS Bioscience Inc. (San Diego, CA, USA) with the following materials: PDE assay buffer (BPS), PDE binding agent (BPS), PDE binding agent diluent for cAMP (BPS), PDE binding agent diluent for cGMP (BPS), Bay 60‐7550 was purchased from Cayman Chemicals (Ann Arbor, MI, USA), and cilostamide, sildenafil citrate and papaverine were purchased from Axxora (San Diego, CA, USA). Bay 73‐6691 was obtained from Sigma‐Aldrich (St. Louis, MO, USA). The assays comprised 10 μm and 1 nm dilutions of the test compound in assay buffer (10% DMSO concentration), and 5 μl of the dilution was added to a 50 μl reaction so that the final concentration of DMSO is 1% in all of reactions. The enzymatic reactions were conducted at room temperature for 60 min in a 50 μl mixture containing PDE assay buffer, 100 nm FAM‐cAMP, or 100 nm FAM‐cGMP, a cnPDE enzyme and the test compound. Bay 60‐7550 (10 μm) was used as a reference compound for PDE1A1, PDE1B, PDE1C and PDE2A1 with respective inhibition of 98%, 97%, 98% and 99%. Cilostamide (10 μm) was used for PDE3A and PDE3B and inhibited 99% and 99% of activity. The reference substance for PDE5 and PDE6C was sildenafil (1 μm) and inhibited both enzymes with 99%. The reference substance for PDE9A was Bay73‐6691 (10 μm) which gave an inhibition of 99%. Papaverine (10 μm) was employed for PDE10A1 and PDE10A2 and inhibited 99% of activity. After the enzymatic reaction, 100 μl of a binding solution (1 : 100 dilution of the binding agent with the binding agent diluent) was added to each reaction, and the reaction was performed at room temperature for 60 min. Fluorescence intensity was measured at an excitation of 485 nm and an emission of 528 nm using a Tecan Infinite M1000 microplate reader. PDE activity assays were performed in duplicate at each concentration. Fluorescence intensity was converted to fluorescence polarisation using the Tecan Magellan6 software. The fluorescence polarisation data were analysed using the computer software, GraphPad Prism (GraphPad Software, San Diego, CA, USA). The fluorescence polarisation (FPt) in absence of the compound in each data set was defined as 100% activity. In the absence of cnPDE and the compound, the value of fluorescent polarisation (FPb) in each data set was defined as 0% activity. The per cent activity in the presence of the compound was calculated according to the following equation: % activity = (FP − FPb)/(FPt − FPb) × 100%, where FP = the fluorescence polarisation in the presence of the compound.

### IC_50_ assay for PDE5A1 characterisation

Phosphodiesterase 5A1 human, recombinant, expressed in Sf9 cells, Supelco Discovery SPE (1 ml) with DSC‐SAX (100 mg/ml), unlabelled cGMP, crotalus atrox venom and bovine serum albumin were purchased from Sigma‐Aldrich. [^3^H]‐cGMP (sp. act 1 mC_i_/mmol) was obtained from PerkinElmer Inc (Boston, MA, USA). The *K*_m_ values of the PDE5A1 cGMP hydrolysis were determined using mixtures of [^3^H]‐cGMP and non‐labelled cGMP to achieve total cGMP concentrations from 0.1 to 10 μm. To obtain IC_50_ values, seven concentrations (0.01 nm–10 μm) of each inhibitor were incubated with 5 μm [^3^H]‐cGMP/unlabelled cGMP. The reaction mixture comprised 20 mm Tris–HCl (pH 7.5), 0.3 mg/ml BSA, 1.5 mm dithiothreitol and 3 mm MgCl_2_. Incubation time was 10 min at 30 °C. In all studies, less than 10% of added [^3^H]‐cGMP was hydrolysed to [^3^H]‐GMP during the reaction. The reaction was terminated by transferring the reactant to a water bath (100 °C for 1 min) and cooled on ice (1–2 min). [^3^H]‐GMP was hydrolysed to [^3^H]‐guanosine by adding 2.5 μl 10 mg/ml crotalus atrox snake venom which contains a potent 5′‐nucleotidase.^11^ The mixture was incubated for 10 min at 30 °C and then diluted in 250 μl 10 mm Tris/8.2 mm propionic acid (pH 7.5). The samples were applied on a preconditioned/equilibrated DSC‐SAX column to separate [^3^H]‐guanosine from [^3^H]‐GMP. The columns were washed with 100 μl 10 mm Tris/8.2 mm propionic acid buffer (pH 7.5) five times. The eluate (400 μl) was transferred to a scintillation vial containing 10‐ml scintillation cocktail (Ultima Gold XR; Packard, Groningen, the Netherlands), and radioactivity was quantified in a Packard 1900 TR Liquid Scintillation analyser.

### Data analysis and statistics

Values for *K*_m_ and IC_50_ were obtained according to Chou,^12^ and *K*_i_ values were calculated according to Cheng and Prusoff.^13^ The descriptive statistics is presented as mean value ± SE in text, tables and figures. Kruskal‐Wallis test (nonparametric ANOVA) with Dunn's multiple comparisons post‐test was used to compare the *K*_i_ values for the analogues with that of sildenafil (InStat, ver. 3.10 for Windows; GraphPad Software).

### Molecular modelling

Docking studies and scoring were performed using the ICM molecular modelling software (Molsoft LLC, San Diego, CA, USA).^14^ The crystal structure of PDE5A1^15^ in complex with sildenafil (PDB ID: 2H42) with the resolution 2.3 Å was converted to an ICM object, and receptor maps were calculated based on the pocket defined by position of co‐crystallised sildenafil in the crystal structure. Ligands were prepared in the ICM ligand editor and converted to 3D when setting up the ligand during the docking session. Charges were also assigned in this step. The ligands were modelled using the ICM molecule editor and docked into PDE5 using interactive docking. Tautomer sampling was performed, as sildenafil may exist in three tautomeric forms. The docking poses were scored by the ICM scoring function. The scoring function gives a score optimised to rank order the docking hits by their binding affinity.^16^ The lower the ICM score, the higher the chance the ligand has a high affinity to the drug target.

## Results

### Sildenafil analogues and cyclic nucleotide phosphodiesterase selectivity

The sildenafil analogues (Table 1) obtained for inhibition studies of cGMP efflux^8^ were screened for their ability to inhibit PDE5A and other members of the cnPDE family (Table 2). As described in methods, the screening was performed with a high and low concentration of the compounds. For the cGMP hydrolysing enzymes, the following order of potency existed; for PDE5A (1 nm inhibitor): IN‐02 = IN‐08 ≥ IN‐01 > IN‐04 > IN‐09 > IN‐11 ≥ IN‐03 = IN‐07 = IN‐05 ≥ IN‐06 = IN‐10. The respective order for PDE6C (1 nm inhibitor) was IN‐01 > IN‐08 = IN‐02 ≥ IN‐04 > IN‐11 > IN‐09 ≥ IN‐03 > IN‐07 > IN‐05 > IN‐06 > IN‐10. The inhibitors had low affinity for PDE9A2 with a test concentration of 1 nm. The members of PDE‐subfamilies tested for cAMP hydrolysis showed negligible inhibition with 1 nm. Increasing the test concentration to 10 μm gave markedly inhibition of some of the other cnPDEs, including the PDE1, PDE2A1 and the PDE3 (sub‐)families. Finally, some of the analogues, in the highest tested concentration, gave virtually complete inhibition of PDE10A1 and PDEA2.

**Table 2:** The inhibitors (IN‐01–IN‐11) listed in Table 1 were screened for their inhibitory activity on a panel of phosphodiesterase (PDE) family members as described in Methods

	PDE1A1	PDE1B	PDE1C	PDE2A1	PDE3A	PDE3B	PD5A	PDE6C	PDE9A2	PDE10A1	PDE10A2
IN‐01	93 ± 1.4	100 ± 2.8	90 ± 0.7	97 ± 0	101 ± 3.5	102 ± 2.1	41 ± 0.7	33 ± 1.4	95 ± 5.7	99 ± 0.7	99 ± 0
	30 ± 1.4	41 ± 2.8	26 ± 0.7	33 ± 0.7	46 ± 2.1	40 ± 0	4.0 ± 1.4	11 ± 1.4	7.0 ± 2.8	39 ± 1.4	30 ± 1.4
IN‐02	94 ± 2.1	96 ± 2.1	97 ± 1.4	97 ± 0.7	98 ± 0	100 ± 4.2	35 ± 1.4	45 ± 2.8	92 ± 0	99 ± 0.7	97 ± 2.1
	44 ± 0	51 ± 1.4	39 ± 1.4	45 ± 1.4	65 ± 1.4	50 ± 2.1	15 ± 2.1	11 ± 2.8	17 ± 1.4	57 ± 0	43 ± 0
IN‐03	96 ± 2.8	98 ± 1.4	98 ± 2.1	95 ± 0	97 ± 0	98 ± 0	93 ± 0.7	69 ± 0	97 ± 5.7	93 ± 0	99 ± 0.7
	2.0 ± 0	6.0 ± 0.7	1.0 ± 0	29 ± 1.4	51 ± 0.7	62 ± 1.4	1.0 ± 0	3.5 ± 0.7	29 ± 2.8	2.0 ± 2.8	7 ± 0
IN‐04	95 ± 3.5	96 ± 0.7	99 ± 0.7	99 ± 0	98 ± 2.1	102 ± 0	49 ± 0.7	49 ± 4.9	95 ± 0	94 ± 2.8	98 ± 1.4
	10 ± 1.4	16 ± 0	0 ± 1.4	46 ± 1.4	50 ± 0.7	65 ± 0.7	1.0 ± 0	4.5 ± 2.1	29 ± 5.7	25 ± 2.1	31 ± 0
IN‐05	94 ± 0	99 ± 2.1	99 ± 0.7	97 ± 1.4	96 ± 0.7	99 ± 1.4	94 ± 3.5	81 ± 2.8	97 ± 3.5	99 ± 0.7	94 ± 4.2
	15 ± 2.1	23 ± 2.1	0.5 ± 0.7	50 ± 1.4	79 ± 0	83 ± 0.7	1.5 ± 0.7	2.5 ± 2.1	37 ± 3.5	46 ± 0.7	53 ± 0
IN‐06	95 ± 0.7	98 ± 3.5	99 ± 0.7	99 ± 1.4	92 ± 0.7	97 ± 1.4	98 ± 2.1	89 ± 2.1	100 ± 3.5	95 ± 4.9	96 ± 0
	2.0 ± 2.8	24 ± 2.8	3.0 ± 1.4	57 ± 2.1	78 ± 0.7	87 ± 2.1	3.5 ± 3.5	6.0 ± 2.8	48 ± 0	52 ± 0.7	63 ± 0
IN‐07	96 ± 2.0	95 ± 3.5	98 ± 0	97 ± 1.4	95 ± 2.1	98 ± 0.7	93 ± 2.1	74 ± 2.1	101 ± 1.4	92 ± 2.8	97 ± 0.7
	12 ± 0	29 ± 1.4	1.0 ± 1.4	60 ± 2.1	73 ± 0.5	83 ± 0.7	2.5 ± 0.7	6.5 ± 2.1	28 ± 3.5	57 ± 0.7	61 ± 1.4
IN‐08	94 ± 0.7	98 ± 2.1	97 ± 0.7	96 ± 1.4	101 ± 2.8	100 ± 0	37 ± 2.8	44 ± 0.7	98 ± 5.7	90 ± 2.8	99 ± 0.7
	16 ± 3.5	22 ± 2.1	2.0 ± 0.7	77 ± 0	78 ± 3.5	84 ± 0.7	1.5 ± 0.7	1.0 ± 0	56 ± 1.4	60 ± 2.8	69 ± 2.1
IN‐09	92 ± 0.7	98 ± 2.1	97 ± 2.1	99 ± 2.1	95 ± 2.8	98 ± 1.4	59 ± 4.2	66 ± 0	100 ± 0.7	98 ± 1.4	97 ± 0.7
	12 ± 1.4	28 ± 0.7	1.0 ± 0	79 ± 2.1	74 ± 0.7	88 ± 2.8	1.5 ± 0.7	1.5 ± 0.7	67 ± 1.4	60 ± 0.7	66 ± 2.8
IN‐10	97 ± 1.4	99 ± 2.1	97 ± 0.7	96 ± 0.7	98 ± 2.1	100 ± 0.7	101 ± 4.2	97 ± 0	95 ± 1.4	94 ± 0.7	101 ± 0.7
	11 ± 0	31 ± 0	3.0 ± 0.7	55 ± 0.7	69 ± 3.5	81 ± 2.1	1.0 ± 0	6.0 ± 1.4	33 ± 4.9	56 ± 4.2	60 ± 0
IN‐11	99 ± 0.7	94 ± 0.7	97 ± 1.4	99 ± 1.4	96 ± 2.1	99 ± 0.7	90 ± 1.4	60 ± 3.5	98 ± 0.7	97 ± 2.8	98 ± 2.8
	11 ± 0.7	28 ± 2.8	5.0 ± 0	34 ± 0.7	83 ± 2.8	91 ± 0.7	4.0 ± 1.4	5.0 ± 1.4	78 ± 0.7	69 ± 3.5	89 ± 2.8

They were tested in duplicates for two concentrations (1 nm/10 μm). FAM‐cAMP (100 nm) was used as substrate for PDE1s, PDE2A1, PDE3s and PDE10s, whereas FAM‐cGMP (100 nm) was used for PDE5A, PDE6C and PDE9A2. PDE5A was also tested with 1 nm and 10 μm sildenafil (as a positive control) and reduced the cGMP hydrolysis to 58 ± 1.4% and 1 ± 0% of control, respectively. Results (mean ± SE) are presented as % of control representing two time‐independent experiments each in duplicate.

### Characterisation of PDE5A1 inhibition by sildenafil analogues

**Figure 1:**
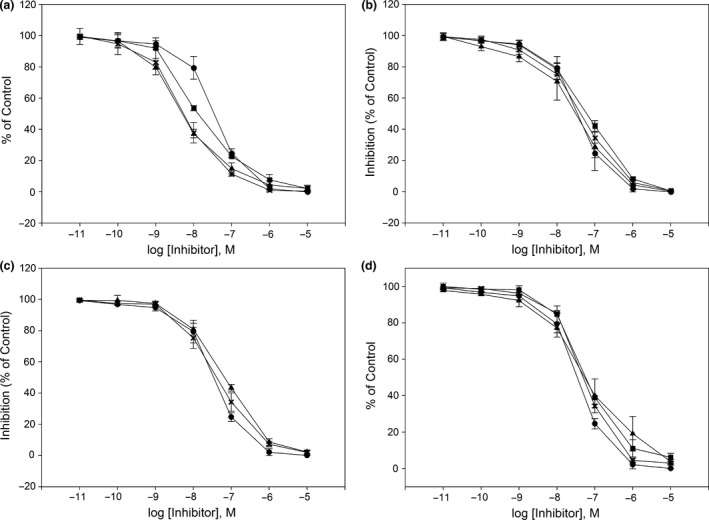
The sildenafil analogues were tested for their inhibition of PDE5A‐mediated cGMP hydrolysis as described in methods. The experimental points represent mean ± SE (*n* = 6). Sildenafil was used as reference substance. Panel a: sildenafil (●), IN‐03 (▲), IN‐08 (×) and IN‐09 (■). Panel b: sildenafil (●), IN‐01 (▲), IN‐02 (×) and IN‐04 (■). Panel c: sildenafil (●), IN‐05 (▲), IN‐11 (×), panel d: sildenafil (●), IN‐06 (▲), IN‐07 (×) and IN‐10 (■).

The characteristics (IC_50_/*K*_i_ values) of the 11 sildenafil analogues were assessed by full concentration–inhibition curves for their ability to inhibit PDE5A1‐mediated cGMP hydrolysis. The *K*_m_ value of PDE5A1‐mediated cGMP hydrolysis was 1.7 ± 0.4 μm. Sildenafil was employed as reference compound for the inhibitors. A *K*_i_ value of 3.3 ± 0.9 nm was obtained for sildenafil under the present experimental conditions. All analogues inhibited the PDE5A1‐dependent cGMP hydrolysis in a concentration‐dependent manner. Figure 1 shows IC_50_ curves for the analogues with sildenafil as reference substance. Table 3 shows both IC_50_ and *K*_i_ values. Three analogues were more potent than the rest (Figure 1, panel a), IN‐03, IN‐08 and IN‐09 with *K*_i_ values from 1.2 to 1.9 nm (Table 3). Figure 1 (panel b) shows the second group (IN‐01, IN‐02 and IN‐04) with intermediate affinities (*K*_i_ values from 2.6 to 3.0 nm), virtually identical with that of sildenafil (Table 3). The third group (Figure 1, panel c) comprised IN‐05 and IN‐11 (*K*_i_‐values were 7.8 and 9.8 nm). The last group (Figure 1, panel d) with the lowest affinities (*K*_i_ value range was 12–14 nm) consisted of IN‐06, IN‐07 and IN‐10. The inhibition curves of sildenafil were shifted from the right (Figure 1, panel a) to the left (Figure 1, panel d). Statistical analysis with Kruskal‐Wallis test (nonparametric ANOVA) gave a P value < 0.0001, considered extremely significant. However, the Dunn's multiple comparisons post‐test showed that only IN‐06 and IN‐10 had Ki‐values significantly different from that of sildenafil (Table 3).

**Table 3:** IC_50_ and *K*_i_ values of PDE5A cGMP hydrolysis for sildenafil and its analogues

Inhibitor	IC_50_ (nm)	*K*_i_ (nm)
Sildenafil	10.3 ± 2.8	3.3 ± 0.9
IN‐01	9.7 ± 0.02	3.0 ± 0.01^ns^
IN‐02	8.2 ± 3.7	2.6 ± 1.2^ns^
IN‐03	4.5 ± 1.3	1.4 ± 0.4^ns^
IN‐04	9.2 ± 0.7	2.9 ± 0.2^ns^
IN‐05	24.8 ± 1.2	7.8 ± 0.4^ns^
IN‐06	44.3 ± 4.9	14.0 ± 1.6*
IN‐07	37.9 ± 6.8	12.0 ± 2.2^ns^
IN‐08	3.9 ± 0.7	1.2 ± 0.2^ns^
IN‐09	6.0 ± 1.2	1.9 ± 0.4*
IN‐10	43.4 ± 2.3	13.7 ± 0.7*
IN‐11	30.5 ± 1.7	9.6 ± 0.6^ns^

IC_50_ curves were obtained for inhibitor concentrations between 0.1 nm and 10 μm, and the IC_50_ value were calculated (given as mean ± SE, *n* = 6) as described by Chou^12^ and transformed to *K*_i_ values (given as mean ± SE) according to Cheng and Prusoff.^13^ The results were obtained from three time‐independent series. The *K*_i_ values of analogues were compared statistically with that of sildenafil. Kruskal‐Wallis test with Dunn's multiple comparisons post‐test; ns = p > 0.05, * = p < 0.05.

### Docking of novel sildenafil analogues to the crystal structure of PDE5A1 catalytic domain

**Figure 2:**
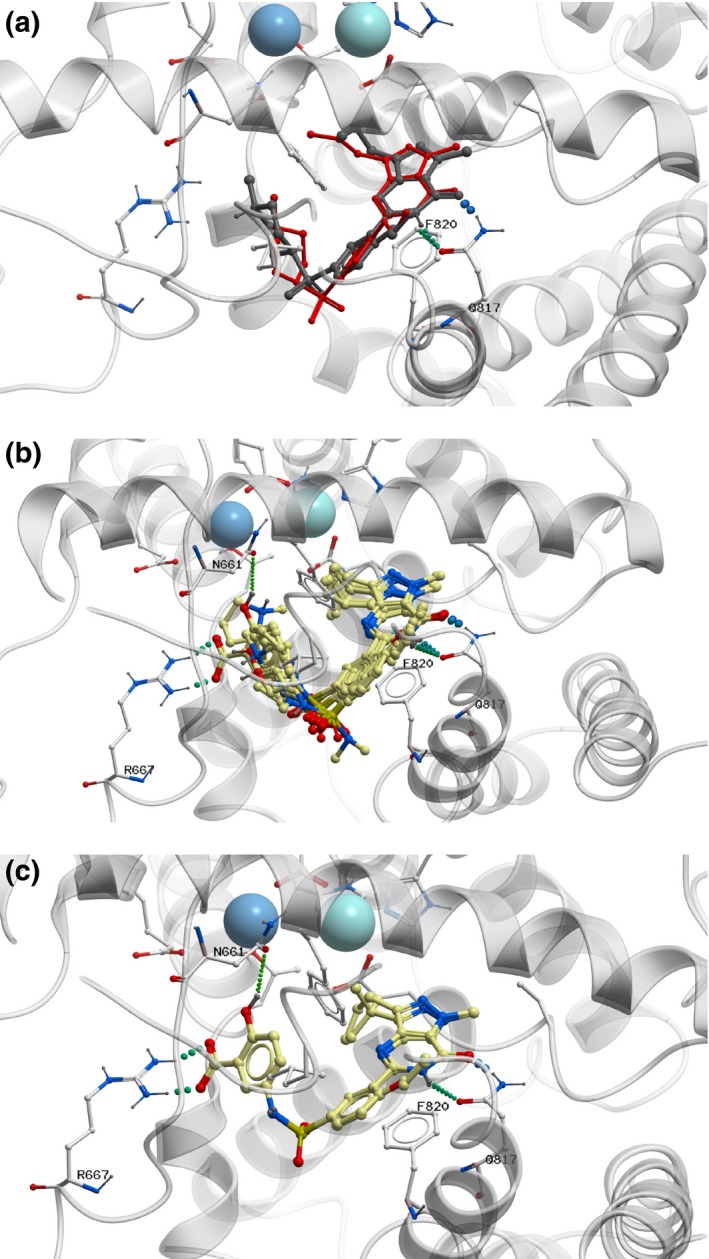
Docking of sildenafil and sildenafil analogues into the crystal structure of PDE5A. Panel a: Location of sildenafil (red) after self‐docking and that of sildenafil (black) co‐crystallised with PDE5. Panel b: Location and poses of the 11 sildenafil analogues in the sildenafil binding site. Panel c: Location of the salicylic acid moiety of the compounds IN‐01 and 1N‐02. [Colour figure can be viewed at wileyonlinelibrary.com]

To assess the accuracy of ICM docking procedure, the sildenafil molecule was docked in the structure of PDE5A1 co‐crystallised with sildenafil. Self‐docking showed that it occupied spatially the same place as sildenafil from crystal structure (Figure 2, panel a). All the analogues had similar binding poses as sildenafil (Figure 2, panel b). The heterocyclic ring system of the sildenafil‐like compounds spatially occupied the same position as in the crystal structure of PDE5A1 with sildenafil. Additionally, the salicylic acid moiety of the compounds IN‐01 and IN‐02 formed hydrogen bonds with Arg667 and Asn661 (Figure 2, panel c).

## Discussion

Sildenafil has become a reference substance for inhibitors of PDE5.^17^ However, other molecular targets for sildenafil have been discovered such as ABC‐transporters. Sildenafil interacts with ABCB1 (P‐glycoprotein) and ABCG2 (breast cancer resistance protein),^18^ ABCC4 (MRP4),^19^, ^20^, ^21^ ABCC5 (MRP5)^3^, ^5^, ^19^ and ABCC10 (MRP7).^22^, ^23^ It was not surprising that the sildenafil analogues identified with VLS^8^ inhibited activity of ABCC5^8^ and ABCC4.^21^ These analogues were characterised in this study to decide whether they were able to distinguish between the various cnPDEs and to determine their affinities for PDE5.

The properties of cnPDEs have been extensively reviewed by Conti and Beavo.^2^ Some PDEs hydrolyse both cAMP and cGMP at low substrate levels (PDEs 1, 2, 3, 10 and 11). The present results showed that members of PDE family 1, 2, 3 and 10 were not, or only to a minor extent, inhibited when analogue concentrations were 1 nm. Increasing the concentrations to 10 μm caused a clear inhibition. Some PDEs selectively recognise and hydrolyse cGMP (PDEs 5, 6 and 9). In this study, the same analogues showed equipotent inhibition of PDE5A and PDE6C. In agreement with this, PDE6 binds sildenafil with similar affinity as PDE5.^24^ On the other hand, the inhibitory potency of PDE9A was much lower.

The screening was succeeded by a thorough characterisation of the inhibitors on PDE5A‐mediated cGMP hydrolysis activity. The two different methods employed for screening and PDE5A characterisation gave similar but not identical results. The methods employed for detailed studies on PDE5A were established 4–5 decades ago^25^ but is still in use after continuous refinements.^26^ The *K*_m_ value of PDE5A cGMP hydrolysis was 1.7 μm, virtually identical to that reported (2 μm) by Francis *et al*.^27^ Furthermore, the *K*_i_ value (3.3 nm) obtained here for sildenafil inhibition of PDE5A is in close agreement with that (4 nm) considered as typical.^27^ The 11 sildenafil analogues were able to inhibit cGMP hydrolysis by PDE5 within a relative narrow range of *K*_i_ values (1.2–14 nm). Only two of the analogues had Ki‐values statistically different from that of sildenafil. This shows that VLS, at least in our hands, is a robust method to predict drug analogues.^8^ The molecular modelling employed in this study emphasises the potential of this technology. The inhibitors were recognised by the same binding site as sildenafil and showed an overlapping interaction. The pyrazolopyrimidine group stacked against phenylalanine‐820 and the compounds formed two hydrogen bonds with glutamine‐817, previously shown to play key role in PDE5 inhibitor binding.^28^

The aim of our work was to identify and characterise sildenafil‐like inhibitors with a balanced effect on cGMP hydrolysis and cGMP efflux. In our experimental set‐ups, we found virtually identical values *K*_m_ values for PDE5 cGMP hydrolysis (1.7 μm) in the present work and *K*_m_ values for high‐affinity cGMP transport as reported in previous studies: 2.4,^7^ 2.2^8^ and 2.6 μm.^21^ On the other hand, the *K*_i_ values of sildenafil inhibition of PDE5A1 cGMP hydrolysis and high‐affinity cGMP efflux are extremely different. Previously we have reported *K*_i_ values of 1.2–3.6 μm 5, 8 for the active cellular extrusion of cGMP. This means a *K*_i_ ratio (transport/hydrolysis) of approximately 1000 : 1. It is intriguing that the *K*_i_ ratios were clearly lower for some of the sildenafil analogues, being ≈25 : 1 for both IN‐01 and IN‐02, and ≈100 : 1 for IN‐03. This demonstrates that single molecules may balance action on these two different molecular targets and thereby enhancing the effect of intracellular cGMP. However, the ABC‐transporters are multipurpose pumps (‘vacuum cleaners’) with the ability to remove excess of potential harmful endo‐ and exobiotics. Development of dual and balanced inhibitors should not completely block this vital cellular function.

## Conclusions

Virtual ligand screening was employed to identify sildenafil analogues. In previous studies, some of these compounds reduced cellular efflux of cGMP. In the present work, several of the analogues were more potent, equipotent or less potent than sildenafil in their inhibition of PDE5A‐mediated cGMP hydrolysis. Taken together, these results demonstrate that it is possible to design inhibitors with dual and balanced action.

## Declarations

### Conflict of interest

The Authors declare that they have no conflict of interests to disclose.

## References

[1] Das A *et al* PDE5 inhibitors as therapeutics for heart disease, diabetes and cancer. Pharmacol Ther 2015; 147: 12–21. 25444755 10.1016/j.pharmthera.2014.10.003 PMC4494657

[2] Conti M , Beavo J . Biochemistry and physiology of cyclic nucleotide phosphodiesterases: essential components in cyclic nucleotide signaling. Annu Rev Biochem 2007; 76: 481–511. 17376027 10.1146/annurev.biochem.76.060305.150444

[3] Jedlitschky G *et al* The multidrug resistance protein 5 functions as an ATP‐dependent export pump for cyclic nucleotides. J Biol Chem 2000; 275: 30069–30074. 10893247 10.1074/jbc.M005463200

[4] Corbin JD *et al* Phosphodiesterase type 5 as a pharmacologic target in erectile dysfunction. Urology 2002; 60: 4–11. 10.1016/s0090-4295(02)01686-2 12414329

[5] Sundkvist E *et al* Pharmacological characterization of the ATP‐dependent low K(m) guanosine 3′,5′‐cyclic monophosphate (cGMP) transporter in human erythrocytes. Biochem Pharmacol 2002; 63: 945–949. 11911846 10.1016/s0006-2952(01)00940-6

[6] Aronsen L *et al* Modulation of high affinity ATP‐dependent cyclic nucleotide transporters by specific and non‐specific cyclic nucleotide phosphodiesterase inhibitors. Eur J Pharmacol 2014; 745: 249–253. 25445042 10.1016/j.ejphar.2014.10.051

[7] Schultz C *et al* Cyclic AMP stimulates the cyclic GMP egression pump in human erythrocytes: effects of probenecid, verapamil, progesterone, theophylline, IBMX, forskolin, and cyclic AMP on cyclic GMP uptake and association to inside‐out vesicles. Biochemistry 1998; 37: 1161–1166. 9454609 10.1021/bi9713409

[8] Sager G *et al* Novel cGMP efflux inhibitors – identified by virtual ligand screening (VLS) and confirmed by experimental studies. J Med Chem 2012; 55: 3049–3057. 22380603 10.1021/jm2014666 PMC4181661

[9] Flores Toque HA *et al* Synthesis and pharmacological evaluations of sildenafil analogues for treatment of erectile dysfunction. J Med Chem 2008; 51: 2807–2815. 18393409 10.1021/jm701400r

[10] Latli B *et al* Novel and potent 6‐chloro‐3‐pyridinyl ligands for the alpha4beta2 neuronal nicotinic acetylcholine receptor. J Med Chem 1999; 42: 2227–2234. 10377228 10.1021/jm980721x

[11] Butcher RW , Sutherland EW . Adenosine 3′,5′‐phosphate in biological materials. I. Purification and properties of cyclic 3′,5′‐nucleotide phosphodiesterase and use of this enzyme to characterize adenosine 3′,5′‐phosphate in human urine. J Biol Chem 1962; 237: 1244–1250. 13875173

[12] Chou TC . Derivation and properties of Michaelis‐Menten type and Hill type equations for reference ligands. J Theor Biol 1976; 39: 253–276. 10.1016/0022-5193(76)90169-7 957690

[13] Cheng YC , Prusoff WH . Relationship between the inhibition constant (KI) and the concentration of inhibitor which causes 50 per cent inhibition (IC50) of an enzymatic reaction. Biochem Pharmacol 1973; 22: 3099–3108. 4202581 10.1016/0006-2952(73)90196-2

[14] Abagyan R *et al* ICM – a new method for protein modeling and design. Applications to docking and structure prediction from the distorted native conformation. J Comput Chem 1994; 15: 488–506.

[15] Wang H *et al* Multiple conformations of phosphodiesterase‐5: implications for enzyme function and drug development. J Biol Chem 2006; 281: 21469–21479. 16735511 10.1074/jbc.M512527200

[16] Totrov M , Abagyan R . Derivation of sensitive discrimination potential for virtual ligand screening In: Istrail S, Pevzner P, Waterman M, eds. Proceedings of the Third Annual International Conference on Computational Molecular Biology. Lyon, France: ACM (New York), 1999: 312–320.

[17] Francis SH *et al* Inhibition of cyclic nucleotide phosphodiesterases by methylxanthines and related compounds In: Fredholm BB, ed. Handb Exp Pharmacol. Berlin Heidelberg: Springer Verlag, 2011: 93–133. 10.1007/978-3-642-13443-2_4 20859794

[18] Shi Z *et al* Sildenafil reverses ABCB1‐ and ABCG2‐mediated chemotherapeutic drug resistance. Cancer Res 2011; 71: 3029–3041. 21402712 10.1158/0008-5472.CAN-10-3820 PMC3078184

[19] Chen ZS *et al* Analysis of methotrexate and folate transport by multidrug resistance protein 4 (ABCC4): MRP4 is a component of the methotrexate efflux system. Cancer Res 2002; 62: 3144–3150. 12036927

[20] Reid G *et al* Characterization of the transport of nucleoside analog drugs by the human multidrug resistance proteins MRP4 and MRP5. Mol Pharmacol 2003; 63: 1094–1103. 12695538 10.1124/mol.63.5.1094

[21] Orvoll E *et al* Misoprostol and the sildenafil analog (PHAR‐0099048) modulate cellular efflux of cAMP and cGMP differently. Pharmacol Pharm 2013; 4: 104–109.

[22] Chen ZS *et al* Characterization of the transport properties of human multidrug resistance protein 7 (MRP7, ABCC10). Mol Pharmacol 2003; 63: 351–358. 12527806 10.1124/mol.63.2.351

[23] Chen JJ *et al* PDE5 inhibitors, sildenafil and vardenafil, reverse multidrug resistance by inhibiting the efflux function of multidrug resistance protein 7 (ATP‐binding Cassette C10) transporter. Cancer Sci 2012; 103: 1531–1537. 22578167 10.1111/j.1349-7006.2012.02328.x PMC3407321

[24] Zhang X *et al* Efficacy and selectivity of phosphodiesterase‐targeted drugs in inhibiting photoreceptor phosphodiesterase (PDE6) in retinal photoreceptors. Invest Ophthalmol Vis Sci 2005; 46: 3060–3066. 16123402 10.1167/iovs.05-0257 PMC1343468

[25] Beavo JA *et al* Hydrolysis of cyclic guanosine and adenosine 3′,5′‐monophosphates by rat and bovine tissues. J Biol Chem 1970; 245: 5649–5655. 4319563

[26] Sonnenburg WK *et al* Identification, quantitation, and cellular localization of PDE1 calmodulin‐stimulated cyclic nucleotide phosphodiesterases. Methods 1998; 14: 3–19. 9500854 10.1006/meth.1997.0561

[27] Francis SH *et al* Cyclic nucleotide phosphodiesterases: relating structure and function. Prog Nucleic Acid Res Mol Biol 2001; 65: 1–52. 11008484 10.1016/s0079-6603(00)65001-8

[28] Zoraghi R *et al* Phosphodiesterase‐5 Gln817 is critical for cGMP, vardenafil, or sildenafil affinity: its orientation impacts cGMP but not cAMP affinity. J Biol Chem 2006; 281: 5553–5558. 16407275 10.1074/jbc.M510372200

